# An intelligent digital twin framework with AI-driven optimization for patient flow and clinical scheduling in smart healthcare systems

**DOI:** 10.3389/fdgth.2026.1835028

**Published:** 2026-07-15

**Authors:** Stalin Victor Balthasar, Suguna Marappan, Logesh Ravi

**Affiliations:** 1 School of Computer Science and Engineering, Vellore Institute of Technology, Chennai, India; 2 Centre for Advanced Data Science, Vellore Institute of Technology, Chennai, India

**Keywords:** AI-enabled digital twin, behavioral consistency validation, clinical scheduling optimization, emergency department operations, patient flow modeling

## Abstract

The operation of the emergency departments at hospitals can be faced with many operational difficulties due to their unpredictable nature, resource scarcity, and increased pressure on services. In this paper, we propose a multi-level AI-enhanced digital twin framework to analyze the patterns of patient flow and clinical scheduling through a case study conducted with the help of three real-life datasets. Our framework incorporates three levels, namely temporal forecasting, clinical robustness, and outcome grounding, to generate an indication about hospital dynamics from real-life datasets. Temporal intelligence level incorporates an LSTM-based model that has shown competitive prediction performance (R² = 0.6785, MAE = 0.0895, RMSE = 0.1103). In addition, our clinical robustness level uses a triage dataset consisting of 560k records to generate an understanding of congestion pattern based on acuity and reveals that maximum congestion is observed at times ranging from 11:00 to 14:00 caused mainly by moderate-acuity patients (ESI-3). Results Layer. The results layer demonstrates the effect of the model, which shows that the mean waiting time is about 35 min. In addition, there is a negative association between the two variables—waiting time and patient satisfaction. Trend analysis across datasets reveals similarities in temporal trends, implying that the developed framework reflects representative features of the hospital environment. Generally, this study emphasizes the importance of incorporating AI-enabled predictions and digital twin models to aid decision-making concerning the analysis of patients' flow and assessment of scheduling policies.

## 1 Introduction

The current health care delivery systems around the world have been experiencing increased levels of operational problems due to growing patient demand, lack of adequate resources, and increased patient satisfaction demands (1). Hospitals can be viewed as dynamic systems that have patients entering and leaving the hospitals, and there exist different treatment paths depending on the particular patient in addition to unavailable resources (2). These occurrences lead to an increase in patients' waiting times, which leads to an imbalance in the workload among clinicians and departments. Inefficiencies in the healthcare system not only result in customer dissatisfaction but also exert pressure on the clinicians and organizations.

Current hospital management systems with their digital patient records are operating using predefined algorithms and staff experience, thus limiting the process of managing patients' treatment schedules and patient flow (3). Traditional methods of scheduling prove to be highly effective in cases where service time is constant, in addition to stable demand. Such a system cannot be effectively used in managing operational problems that arise due to emergency patient admissions and staffing issues, as well as delays during the treatment process (4).

Firstly, digital twins serve as a practical solution in managing healthcare operations effectively in such an environment. The digital twin in a healthcare context is used to understand operations and user experience in the healthcare system from the digital model (5). In using digital twins, health system administrators will be able to see how the systems work as they run experiments on scenarios without disrupting patient services. Hospitals use the strategy to create a systematic approach that gives insight into patient interaction with clinical services (6).

The use of artificial intelligence makes the digital twin more effective in decision-making. They make use of the ability to predict and adapt as provided for in the digital twin technology (7). This is done by applying the analytical capacity of the AI to predict future patients' numbers and treatment time based on past and current data. The researchers are concerned with creating a clinical scheduling tool that utilizes the predictive analysis of AI and representation technology of digital twins (8). Their findings provide the basis for the creation of an AI-based digital twin for hospitals (9).

**Figure 1:**
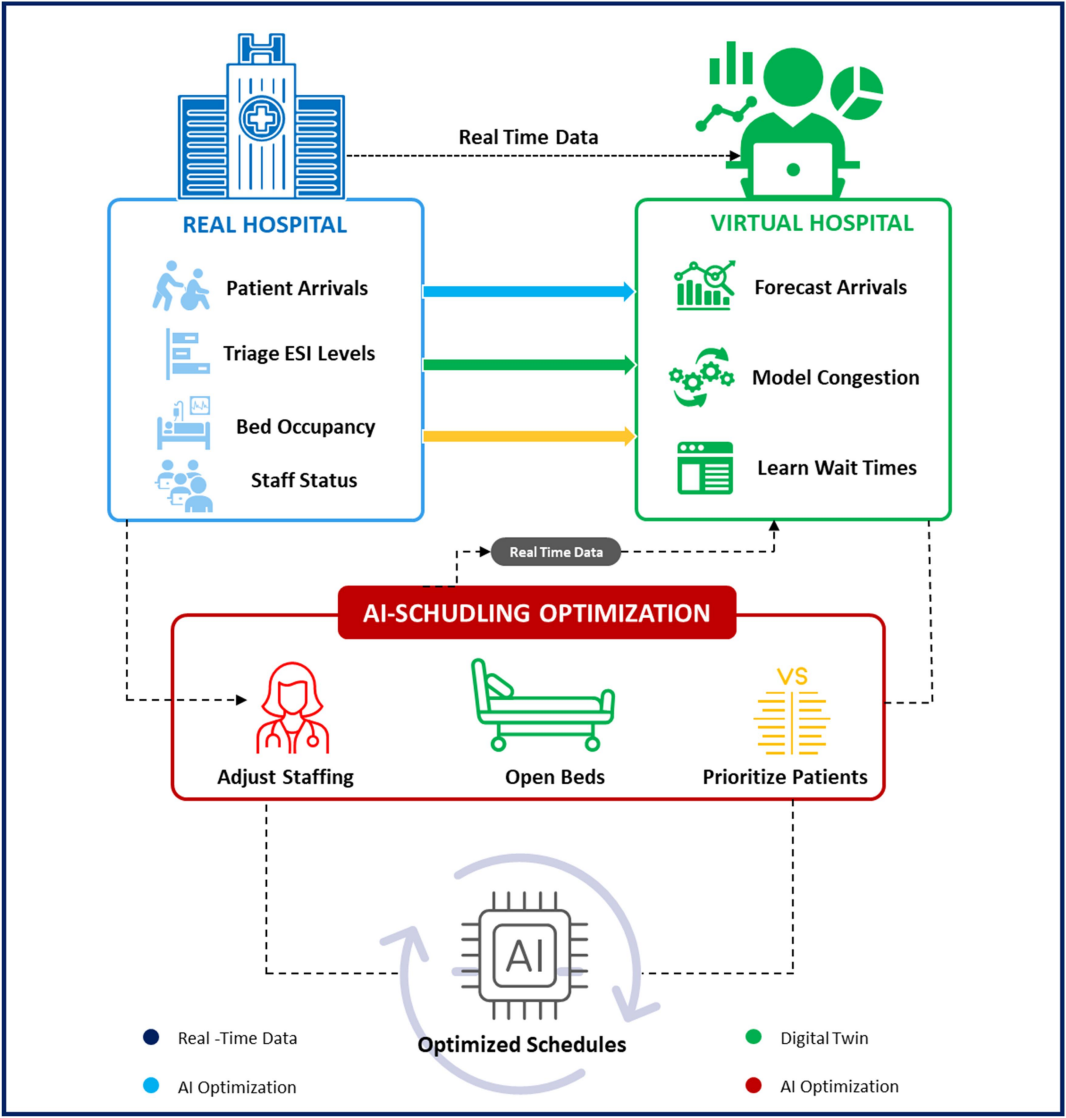
Conceptual overview of an AI-enabled digital twin framework for patient flow and clinical scheduling in hospital environments.

The developed methodology involves using an AI-powered digital twin system for modeling hospital systems, which is shown in Figure 1 below. In other words, the research proposes to develop a conceptual and data-driven digital twin system, which would mirror some of the major aspects of hospital management, including patient flows, resource usage, and scheduling interaction, among others. While not a functioning system in itself, the proposed methodological approach involves creating a virtual model of a real system, which could be employed to test and explore different system properties and possible approaches to its management. The interaction between the digital twin and hospital-related data would allow following patient flows and testing different scheduling scenarios in the process.

This research proposes a digital twin-based framework with AI support for hospital management and clinical scheduling analysis. Specifically, this approach combines predictive models with a virtual environment to simulate the dynamics of patients' movement and resources' interaction within healthcare systems. Compared to predictive models, this framework allows the analysis of several scenarios for assessing the behavior of healthcare systems under different conditions. Artificial Intelligence (AI)-based digital twins combined into one framework allow for decision analysis based on simulations rather than representing an automatic system for operational control. Thus, the proposed framework complies with modern approaches to designing AI-enabled digital twin frameworks for healthcare systems (10).

In this context, the purpose of this research is to propose a digital twin model that can be used to analyze patient arrivals, treatment procedures, and resource allocation using data-driven techniques, specifically predictive analytics. In addition to the ability to forecast uncertain events, the use of digital twin modeling provides the opportunity to examine the impact of patient flows and resource interactions on the operation of the scheduling system by simulating different scenarios. Consequently, this approach enables decision analysis without interfering with real-world processes.

The major contributions of this paper are as follows:

- Multilevel AI-powered digital twin architecture is suggested, which includes temporal prediction, clinical robustness modeling, and outcome grounding in order to comprehensively model emergency department operations.
- A methodology based on data-driven and simulation modeling techniques is designed to analyze patient flow and scheduling without the need to deploy the actual system in real-time.
- Temporal prediction is modeled by means of LSTM to account for medium-term arrival predictions in healthcare settings.
- A congestion modeling methodology that takes into account severity of patients is considered, including different ESI levels in order to mimic the realistic load distribution in an ED.
- A multilevel evaluation technique is introduced, showing consistent behavior of arrivals, congestion, and wait time outcomes to support decision-making.

## 2 Literature review

Advancements in healthcare technology have shown the increasing role of DT and AI in the improvement of health services processes and decision making. Some studies have been conducted on the use of these two technologies in healthcare to make systems more efficient and patient-centered. A summary of related studies on AI and digital twin applications in healthcare operations is presented in Table 1.

**Table 1:** Summary of related studies on AI and digital twin applications in healthcare operations.

Author(s), Year	Study focus	Methodology	Key contribution	Research limitation
Mikołajewska et al. (20)	Patient digital twins in healthcare	AI-enabled CPS and DT frameworks	Highlighted AI-driven digital twins for personalized clinical decision support	Limited clinical validation and fragmented research efforts
Mokhtari, Melvin. (21)	Human Digital Twins in healthcare	Conceptual HDT architecture and networking framework	Introduced HDTs as dynamic virtual replicas, enabling personalized and remote healthcare services	Largely conceptual, with limited empirical validation and practical deployment evidence
MAMTA, and Shravya Reddy KARRI. (22)	Digital twin adoption in healthcare services	Conceptual IoT–AI–Big Data framework	Demonstrated DT applications for monitoring and decision support	Data security, cost, and interoperability challenges
Chen et al. (23)	Human Digital Twins for personalized healthcare	Survey of HDT architecture and networking layers	Defined the HDT framework and its role in personalized healthcare applications	Predominantly conceptual, with unresolved design and implementation challenges
Attaran et al. (24)	Digital Twin applications across industries	Systematic literature review	Reviewed DT evolution, enabling technologies, and applications	Lacks a healthcare-specific and clinical validation focus

### 2.1 Digital twin applications in healthcare

The usage of digital twins has increased significantly to monitor situations and predict outcomes. For example, works by Saratkar et al. (14) and Roopa et al. (16) show how DT can be used in real time to model patients and provide tailored treatments based on the course of their illness. In a similar way, according to Kandan et al. (19), DTs can help in healthcare facility management through real-time monitoring and operation optimization.

### 2.2 AI-driven healthcare systems and predictive modeling

The application of artificial intelligence is vital when dealing with vast healthcare data and performing predictive analytics. According to the work of Gandhi et al. (13) and Shaikh et al. (15), artificial intelligence aids in enhancing clinical decisions, patient stratification, and monitoring through EHRs and wearable technology. Kuppusamy and Palanivel (8) further note that digital twin-based predictive systems powered by artificial intelligence aid in automating healthcare services and enhancing diagnosis precision.

### 2.3 Digital twin and simulation-based healthcare frameworks

A number of research efforts have explored the application of digital twins in simulating and supporting decisions within the healthcare industry. In Rahimi et al. (12), it is pointed out that despite the emergence of healthcare systems employing digital twins, there has been inadequate effort to establish frameworks for evaluation and validation in research. Likewise, Jameil et al. (11) have applied DT in conjunction with edge computing and task offloading.

### 2.4 Research gap

Although there have been substantial improvements, the current research works mostly concentrate on the separate applications of the digital twin concept, like patient tracking, facility management, or predicting events. However, the development of an integrated system that would incorporate temporal predictions, workload modeling, and outcome-driven analysis in a single digital twin remains to be done. Also, the current literature includes very few studies where the proposed methodology was tested with multiple datasets.

In order to bridge the gaps in previous research, we present a novel multi-layer AI-powered digital twin framework with the integration of temporal intelligence, severity-driven congestion, and outcome grounding concepts.

AI and digital twin research in healthcare progresses, yet crucial requirements remain unmet. Recent studies have highlighted the growing role of digital twins and AI-driven analytics in healthcare transformation and operational intelligence (17, 18). Conceptual frameworks, architectural designs, and survey-based assessments dominate AI-enabled digital twin research, not real-time synchronisation and full medical application Rahimi et al. (12), Saratkar, (14). Digital twins may be utilised for personalised therapy, facility management, or computational offloading, although most initiatives concentrate on infrastructure, patient monitoring, or data administration. AI models do not link to digital twins that replicate patient flow and clinical scheduling, therefore they are autonomous prediction and decision-making tools. Lack of full, real-time, and predictive scheduling systems hinders digital twin ideas in proactive healthcare operations. An AI-based digital twin system is needed to create an integrated model that blends patient flow patterns with clinical scheduling systems to assist data-driven hospital decision-making.

## 3 Proposed methodology

This study presents an AI-based digital twin system to simulate hospital patient flow and emergency department clinical scheduling. To enable proactive and data-driven decision-making, the framework uses real-world operational data, a virtual digital twin of hospital operations, and AI-based prediction and optimisation models. The suggested closed-loop system leverages operational data and predictions to maintain digital twin status, allowing realistic simulation and schedule alterations without disrupting ongoing clinical processes.

**Figure 2:**
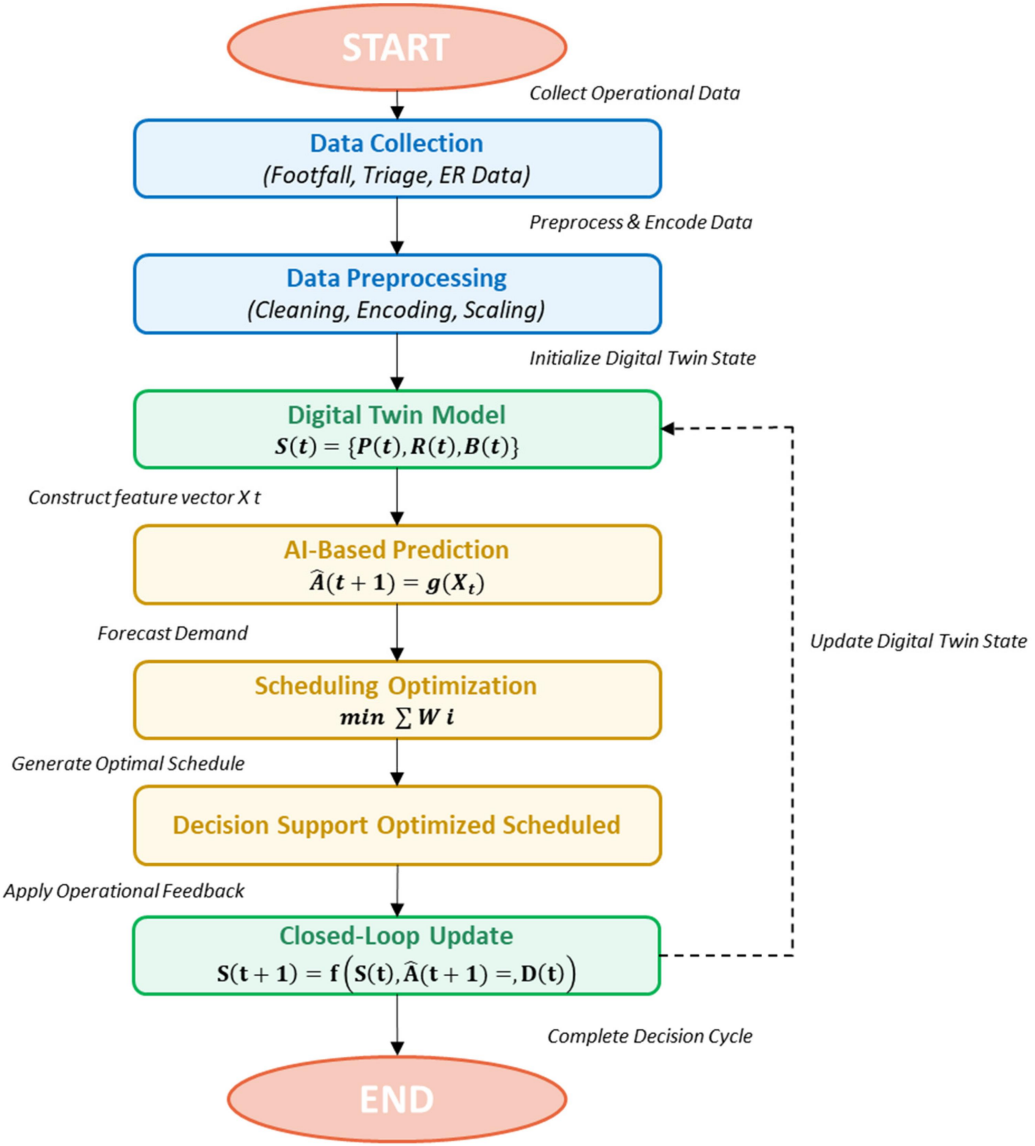
Workflow of the proposed AI-enabled digital twin framework for hospital patient flow and clinical scheduling.

Figure 2 illustrates a closed-loop digital twin workflow in which hospital operational data are used to initialize the system state, AI models predict short-term patient arrivals, and scheduling optimization minimizes patient waiting time. Optimized decisions are fed back in updating the digital twin to enable data-driven clinical scheduling without interrupting any live operations in the hospital.

The methodology is organized into five tightly coupled stages: (i) data collection and preprocessing, (ii) digital twin modeling, (iii) AI-based prediction and optimization, (iv) decision-support and scheduling strategy, and (v) closed-loop workflow and experimental setup. The next section provides a comprehensive explanation of each phase.

### 3.1 Data collection and preprocessing

The AI-enabled digital twin system development tests the three open-access healthcare datasets from Kaggle. The Patient Footfall Prediction Dataset contains two types of data which are time-based arrival projections and actual patient arrival counts recorded at specific times. The Hospital Triage and Patient History Dataset contains data about patient emergency department arrival times and the ESI scores and their medical history which enables experts to create congestion models based on patient severity levels. The researchers use waiting time together with admission result and discharge status information from the Hospital Emergency Dataset to establish outcomes which they use to test operational procedures. The standard preprocessing procedure starts with missing value identification before it proceeds to eliminate data inconsistencies and then it applies numerical feature normalization and categorical variable encoding methods on the dataset which has already been anonymized. The system protects event sequence integrity which enables accurate modeling of patient movement patterns. The processed data supports both digital twin simulation and AI-driven prediction and scheduling modules.

### 3.2 Digital twin modeling approach

The digital twin layer keeps track of patient movements, utilization of resources, and scheduling activities to construct a model for hospital processes. The system state at time t can be stated as follow. The mathematical representation of the proposed digital twin modeling and scheduling framework is presented in Equations (1)–(8).

S(t)={P(t),R(t),B(t)} (1)

P(t) could be considered as patient flow circumstances, while R(t) represents clinical resource availability, and B(t) stands for bed occupancy. The digital twin model changes continuously based on the real-time operational data D(t), which is as follows:

S(t+1)=f(S(t),D(t)) (2)

The digital twin state update process used to update patient flow, resource availability, and bed occupancy within the proposed framework is summarized in Algorithm 1.

Algorithm 1 **Digital Twin State Update Mechanism**

**Input:** Initial system state S(t), real-time operational data D(t)

**Output:** Updated system state S(t+1)

 1. Initialize digital twin with current state S(t)={P(t),R(t),B(t)}

 2. Obtain current operational data D(t).

 3. Update patient flow status P(t + 1) through arrival and completion times.

 4. Update resource availability R(t + 1) through staff usage and system utilization.

 5. Update bed occupancy B(t + 1) through admissions and discharges.

 6. Construct updated system state.

S(t+1)={P(t+1),R(t+1),B(t+1)} (3)

 7. Return updated state S(t+1)

### 3.3 AI model integration for prediction and optimization

The proposed digital twin model combines the use of AI for predicting patient arrivals with an analysis component of the scheduling problem to aid decision-making using simulation techniques. Instead of developing a functional optimization algorithm, the model offers a conceptual framework and data-driven insights to understand how demand predictions could affect scheduling decisions in hospitals.

The hospital system at time t can be modeled as:

$$X_{t}=\{A(t),P(t),R(t),B(t),\tau (t)\}\;$$ (4)

where A(t) is the historical arrival of patients, P(t) is the patient flow status, R(t) is the resource status, B(t) is the bed occupancy status, and *τ*(t) is the time-dependent feature.

The predicted arrival rate for the next time step is determined with the help of an AI model trained by the system as follows:

$$\hat{A}(t+1)=g(X_{t})\;$$ (5)

The forecasted demand becomes part of the digital twin to generate a future system state:

S(t+1)={P(t+1),R(t+1),B(t+1)} (6)

In order to investigate scheduling behavior under predicted demand, the research considers the objective of minimizing total patient waiting time:

$$\min\overset{N}{\underset{i=1}{\sum }}W_{i}$$ (7)

where $W_{i}$ stands for the waiting time of patient i, and N stands for the total number of patients.

In addition, the congestion state can be understood from a severity perspective, where the patient load will be measured by ESI levels. Under this assumption, moderate patients (ESI-3) will carry a greater weight towards the overall congestion state because of their larger numbers, whereas high (ESI-1/2) and low (ESI-4/5) acuity cases will be included according to their appropriate weights. With this severity perspective, the digital twin model can effectively reflect the congestion behavior in real life situations.

In the proposed digital twin model, the decision variable is formulated as conceptual decision variables like staff allocation levels, bed assignments, and service priorities. The scheduling decision variables are subject to practical constraints like available staffs, bed capacity, and service times. Instead of employing an optimizer, the digital twin model will simulate a set of feasible scheduling solutions to observe their impacts on waiting times.

The AI-enabled prediction and scheduling evaluation process is presented in Algorithm 2.

Algorithm 2 **AI-Enabled Prediction and Scheduling Evaluation**

**Input:** Arrival history data, digital twin status at time t S(t), AI model g(⋅)

**Output:** Evaluated scheduling recommendation

 1. Initialize digital twin state S(t)={P(t),R(t),B(t)}

 2. Construct system feature vector $X_{t}=\{A(t),P(t),R(t),B(t),\tau (t)\}$

 3. Predict short-term patient arrivals $\hat{A}(t+1)=g(X_{t})$

 4. Predict future system states based on forecasted arrivals

 5. Determine a set of scheduling alternatives (e.g., staff reassignment, priority shifts, bed relocation)

 6. Test each alternative using the digital twin

 7. On the basis of forecasted waiting timeSelect the scenario with the lowest projected waiting time

 8. Output scheduling recommendation

The functioning of the digital twin system involves the constant combination of prediction results regarding patient flow with the existing state of the system, creating a virtual reality of hospital functioning. On every cycle, the information received from the AI regarding the predicted patient arrivals will be entered into the digital twin, resulting in the change of variables related to patient flow, available resources, and bed occupancy. Depending on these new variables, several scenarios will be analyzed based on staff allocation and resource usage.

The analysis of these scenarios will allow estimating the possible waiting times for patients and determining whether there will be any problems concerning the use of resources and their allocation. This will help to see what consequences can arise depending on the decisions of healthcare administration, making decisions without causing any changes in the actual environment of a hospital.

### 3.4 Decision-support and scheduling strategy

The decision support layer employs simulation techniques that allow for assessing scheduling policies by employing a digital twin. In contrast to the previous layer that ensures real-time implementation of the schedule, the current model is designed to provide hospital administrators with the opportunity to experiment with various scenarios of operation to assess the influence of each on patient queue formation and wait time. The scheduling goal is consistent with the one stated above and involves minimizing total wait time of patients in the emergency room. It is adopted because wait time can be viewed as an indicator of congestion.

In the digital twin environment, different scheduling policies can be implemented, including shifting of staff to other departments, prioritizing certain services, and redistribution of resources. These options will be considered and analyzed in terms of wait time and resource usage. At this stage, it is required to select the most promising scheduling policy from those evaluated. By doing so, it is possible to ensure that the results are understandable and comply with clinical goals.

### 3.5 Workflow of the proposed framework

The closed-loop process adopted by the AI-based digital twin model enables the system predictions to receive operational feedback, resulting in hospital operations synchronization. The digital twin model is updated at each time step t through:

S(t+1)=f(S(t),D(t)) (8)

The current state of the digital twin is denoted as S(t), while the newly acquired operational data D(t) consists of patient arrivals and completion of services, as well as resource consumption information. As hospital settings differ from one another, it becomes necessary to update the digital twin again. The proposed model allows testing different prediction and operational methods in the simulation environment.

All experiments were carried out using Python in the Anaconda Jupyter Notebook on a computer equipped with 8 cores, 16 GB of RAM, and 1 TB of SSD space.

### 3.6 Experimental setup and model configuration

In order to achieve reproducibility, a sequence-based method was used to transform time-series data into supervised learning problem using a sliding window with 30 time-steps that provide temporal information at a monthly level. The final dataset contained input values of (602, 30, 6), where 6 is the feature space. Time series split was performed where 80% of the dataset is used for training while 20% for testing, and further 10% of the training set is considered for validation to avoid overfitting.

A temporal prediction model has been created using LSTM, which stands for long-short term memory. It belongs to the family of recurrent neural networks specifically designed for the analysis of sequential data. In this case, there are two LSTM layers with 64 and 32 units respectively, alongside with an output layer that predicts the target values. The use of a dropout layer with a rate of 0.2 ensures better generalization and reduces overfitting. Training process involved the Adam optimization algorithm with a learning rate of 0.001 and loss function of mean squared error (MSE).

In contrast to CNN-based methodologies, the suggested architecture aims at extracting temporal dependencies from sequential patient admission information as opposed to spatial features. Through its stacking mechanism, the architecture has the capability of learning short-term and long-term temporal dependencies that can help capture dynamics and time dependency in healthcare demand.

Normalization of input features and temporal ordering were performed before training the model. The evaluation of the architecture's predictive performance was done through the application of regression metrics such as MAE, RMSE, and R².

### 3.7 Ethics statement and data governance

The entire study uses datasets that are available to the public and are de-identified as well. There are no personal data included in any part of the analysis, and all the data have been processed in a manner that keeps privacy and confidentiality intact. Since there is no human experimentation or interaction involved in this study and the data are publicly available, there is no need for ethical approval.

## 4 Results and discussion

This part contains the findings of the experiments carried out during the testing of the suggested digital twin approach for analyzing ED operations, based on multiple datasets. These datasets have been employed in the process of studying various components of the system such as patient arrivals, ED congestion, and waiting times. It is not a complete real-life implementation; rather, it can be viewed as a simulation of operations.

### 4.1 Dataset-1: patient footfall prediction results (temporal intelligence layer)

From the performance comparison as displayed in Table 2, it is clear that both linear and ridge regressions have performed very well as the most accurate predictive models from the smoothing exercise carried out on the patient footfall data. On the other hand, the performance of the LSTM model is quite impressive since it has an R² score of 0.6785, while also being able to account for medium-level temporal features within patient arrivals. The reason why the LSTM, Random Forest, and XGBoost have poor performance scores after applying the smoothing exercise is because the temporal complexity of the data is lost in the process, hence, no longer requiring nonlinear modeling. Nonetheless, the LSTM algorithm is still applicable given the fact that it is capable of modeling nonlinear temporal dependencies, especially in an unsmoothed data context.

**Table 2:** Comparative performance evaluation of predictive models on smoothed patient footfall data.

Model	MAE	RMSE	R²
Linear Regression	0.0788	0.0933	**0**.**7703**
Ridge Regression	0.0791	0.0934	0.7698
SVR (RBF Kernel)	0.1077	0.1318	0.5413
Random Forest	0.0861	0.1063	0.7015
XGBoost	0.0950	0.1190	0.6257
**LSTM (Proposed)**	0.0895	0.1103	0.6785

Bold values indicate the best-performing model based on the respective evaluation metric.

**Figure 3:**
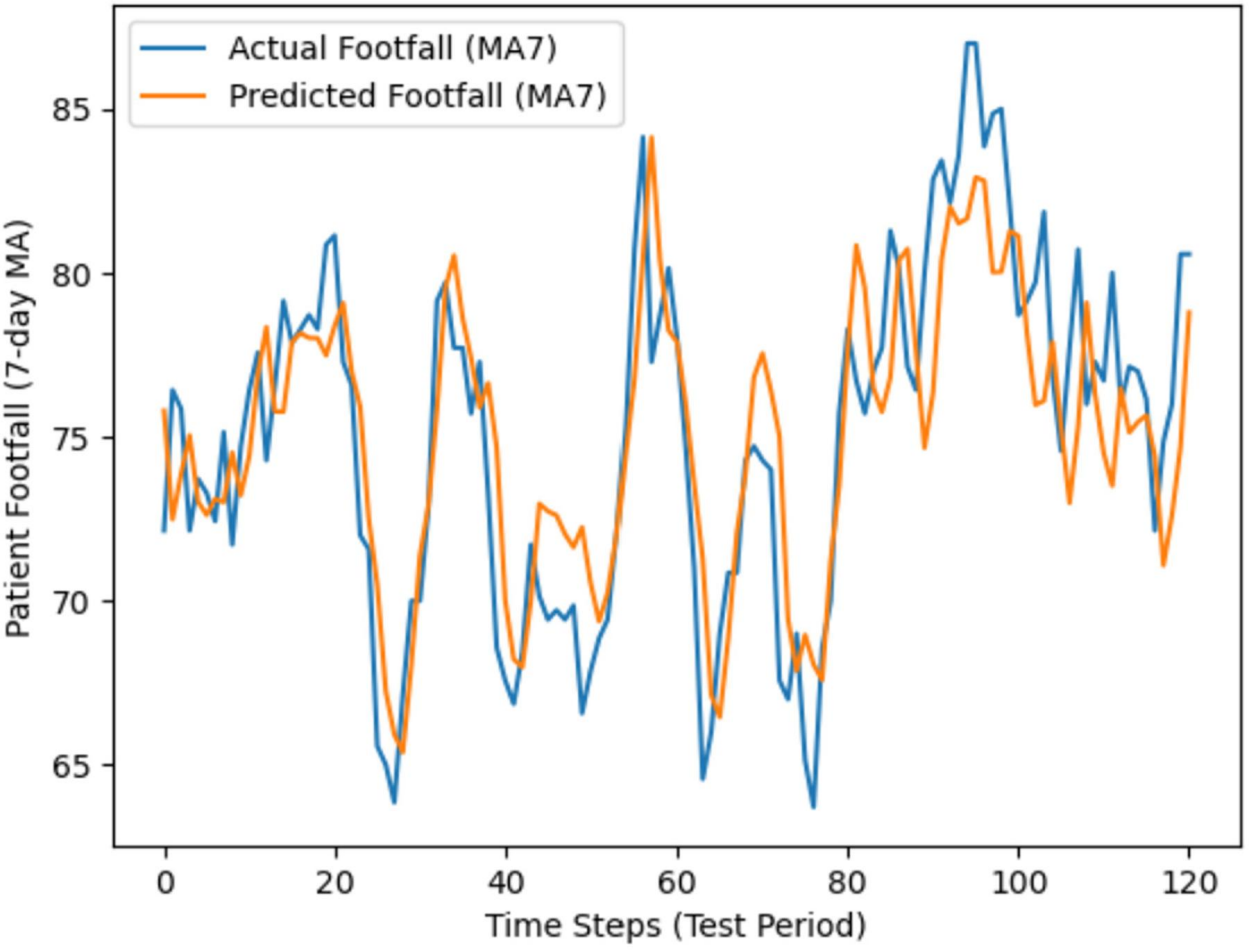
Comparison of predicted and actual smoothed patient footfall (7-Day moving average).

In a study of the seven-day moving average trend of predicted and actual patient arrivals, the LSTM model's ability to imitate actual demand patterns is demonstrated in Figure 3. Since all peaks and valleys of the LSTM model coincide with actual values, the model can predict temporal patterns by using both. As actual arrival patterns cannot be predicted, the model displays minute temporal spaces during fast shifts. Temporal intelligence can predict patient arrivals accurately.

**Figure 4:**
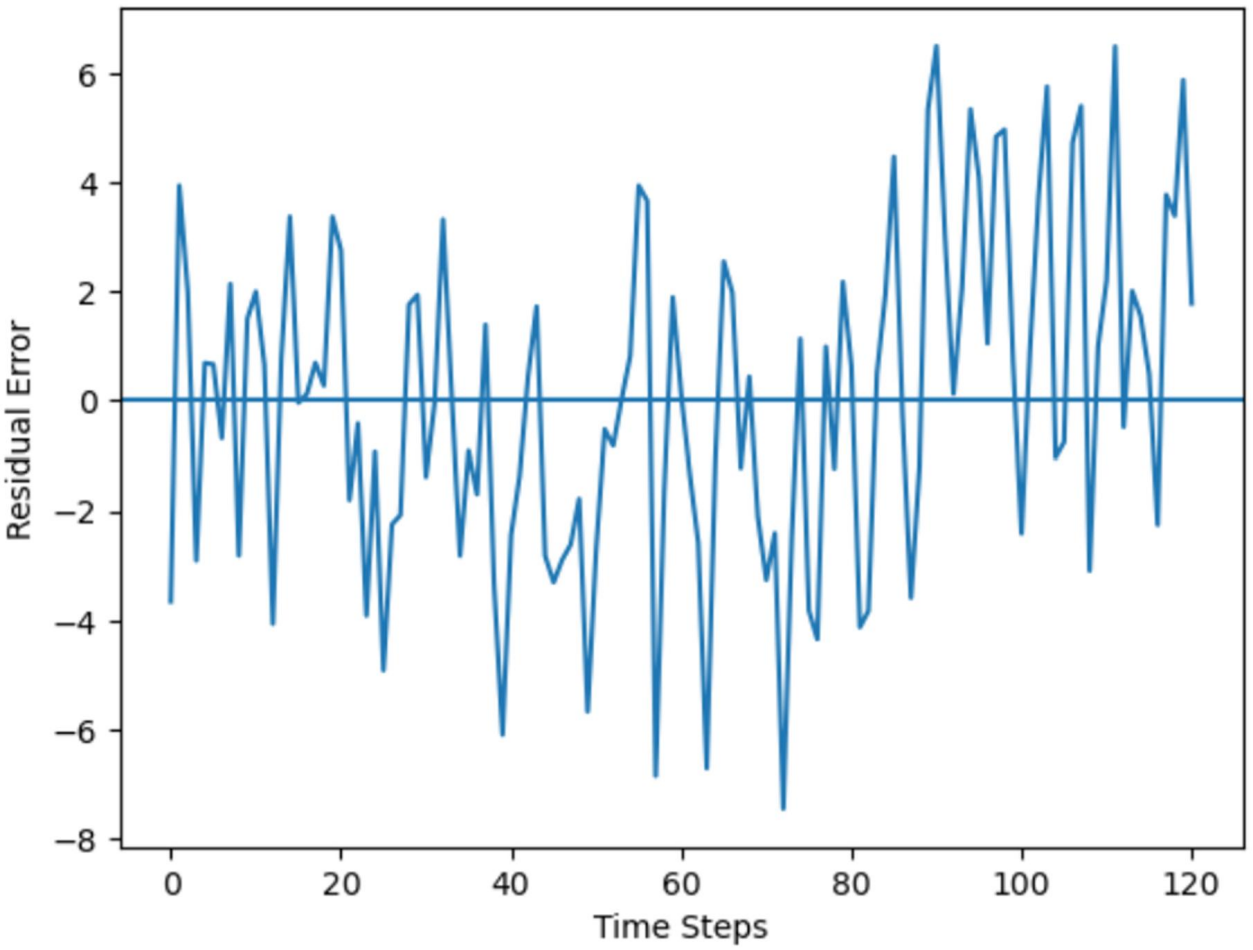
Temporal residual error dynamics of the LSTM-based footfall predictor.

Figure 4 shows the development of the residual errors of the LSTM model. The reason is that the residuals of the model are symmetrical and concentrated around zero. The short-term peaks that disappear after the change in demand aid the model to learn the seasonal and cyclical patterns. The temporal intelligence layer is efficient because it can deal with different demand situations.

**Figure 5:**
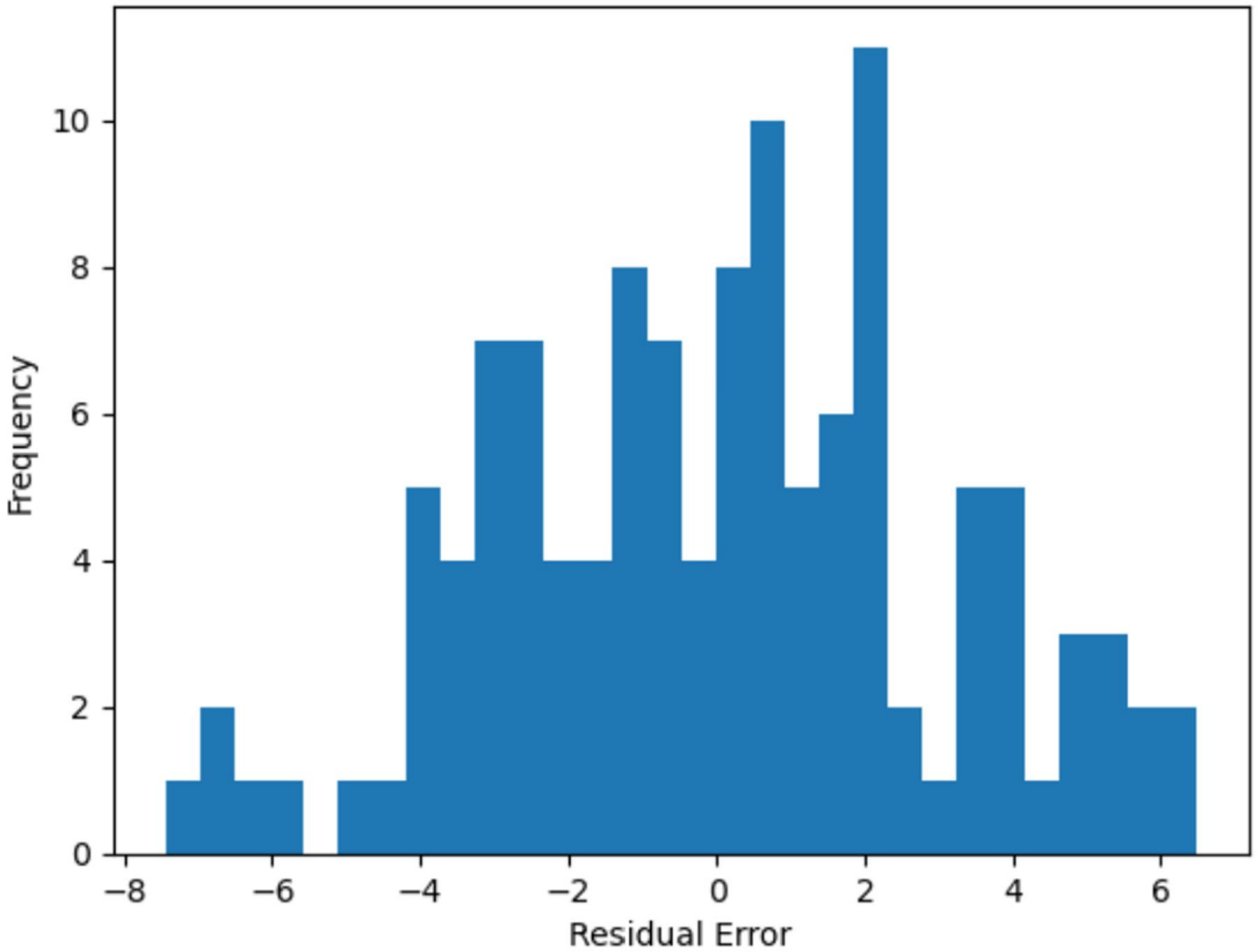
Distribution of prediction residuals for LSTM-based patient footfall forecasting.

The central tendency is very prominent as depicted in Figure 5, where there is symmetry between the prediction residuals. Prediction residuals are unbiased as they do not exceed certain limits. There are no instances of extreme heavy tailed distributions, thus establishing generalisation. Data smoothing is first applied to stabilize time-series data before predicting hospital foot traffic through LSTM architecture.

### 4.2 Dataset-2: clinical robustness and digital twin validation

**Figure 6:**
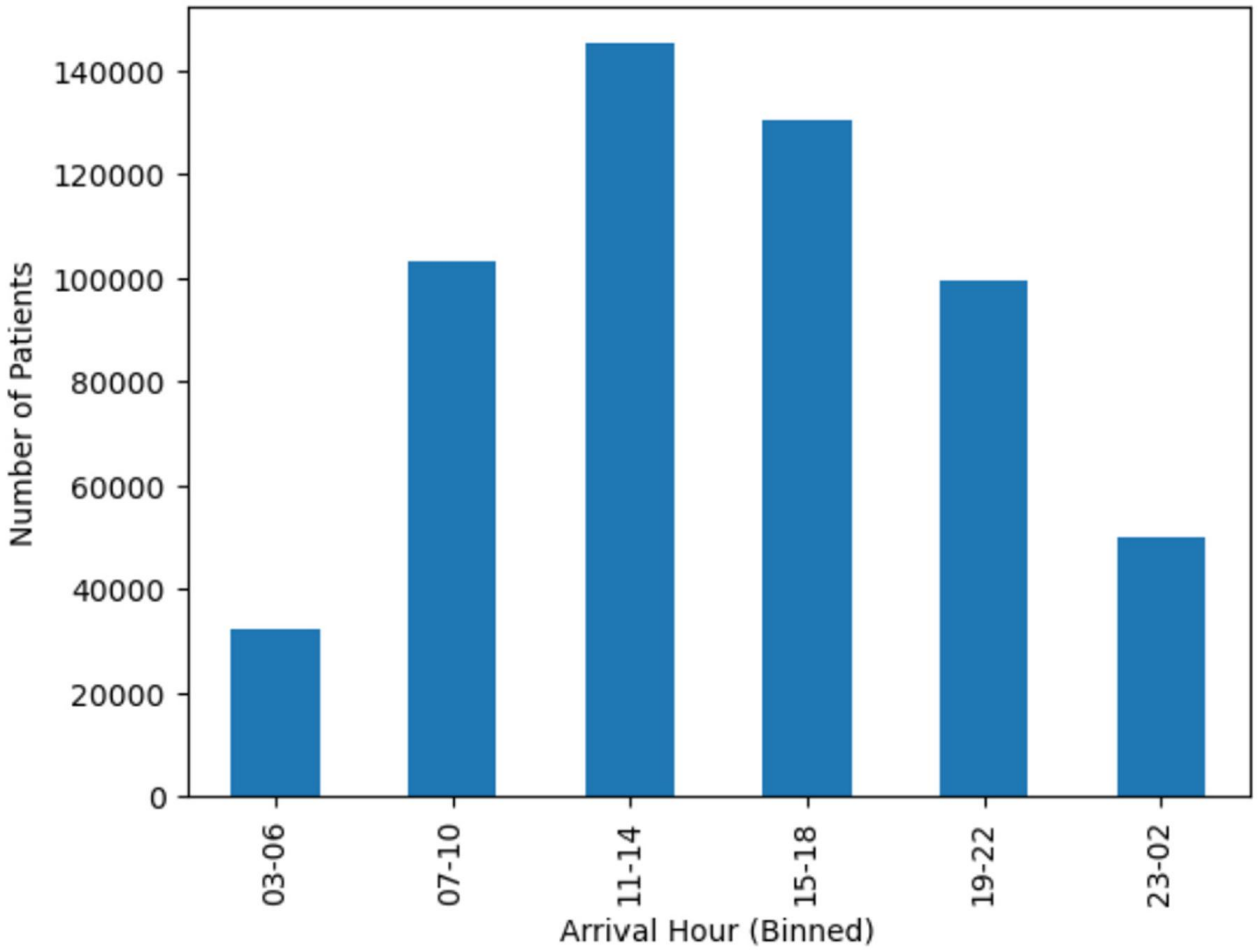
Hourly distribution of emergency department patient arrivals based on triage records.

According to the data obtained from triage in the hospital's emergency room, Figure 6 below shows the distribution of patient arrivals by hour of the day. It has been established that patients tend to come in higher numbers during the intervals of 11:00–14:00 and 15:00–18:00, while there is a considerable reduction in patients coming in at 23:00–06:00. Thus, it can be concluded that there is an organized process of patient arrivals per day, with more arriving in the daytime due to outpatient referrals and diagnostics.

**Figure 7:**
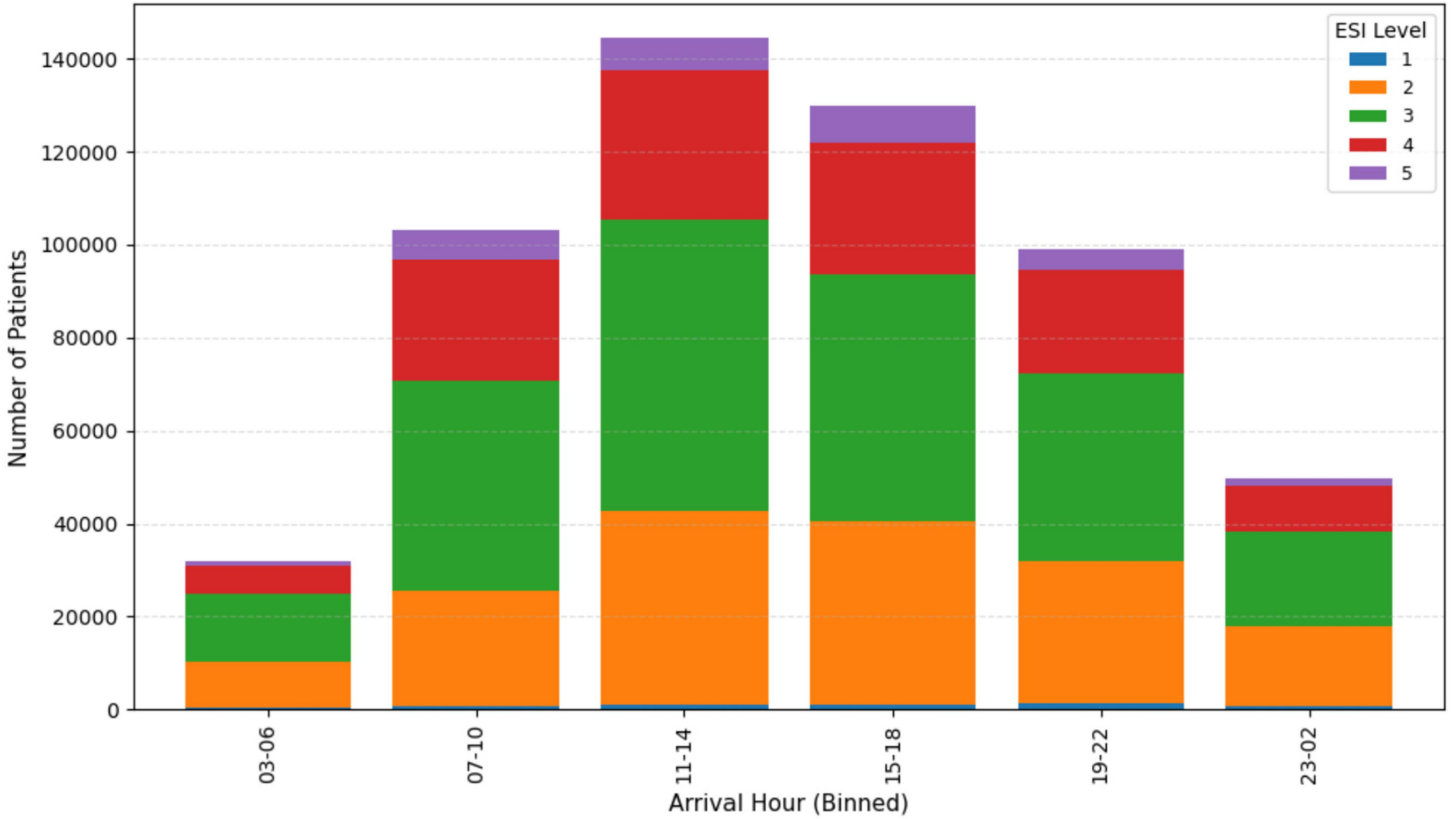
Severity-Stratified patient load across emergency department arrival hours.

The patient arrivals according to severity classification are demonstrated on Figure 7. It can be seen that there is the highest number of arrivals from the category of moderate-severity patients (ESI-3). They appear most often during the period of 11:00–14:00. Fewer high-acuity patients (ESI-1/2) appear at any time of the day, whereas the number of low-acuity patients (ESI-4/5) appears higher at certain times during the day and at the beginning of the evening. This demonstrates the effect that the number of moderate-severity patients has on congestion.

**Figure 8:**
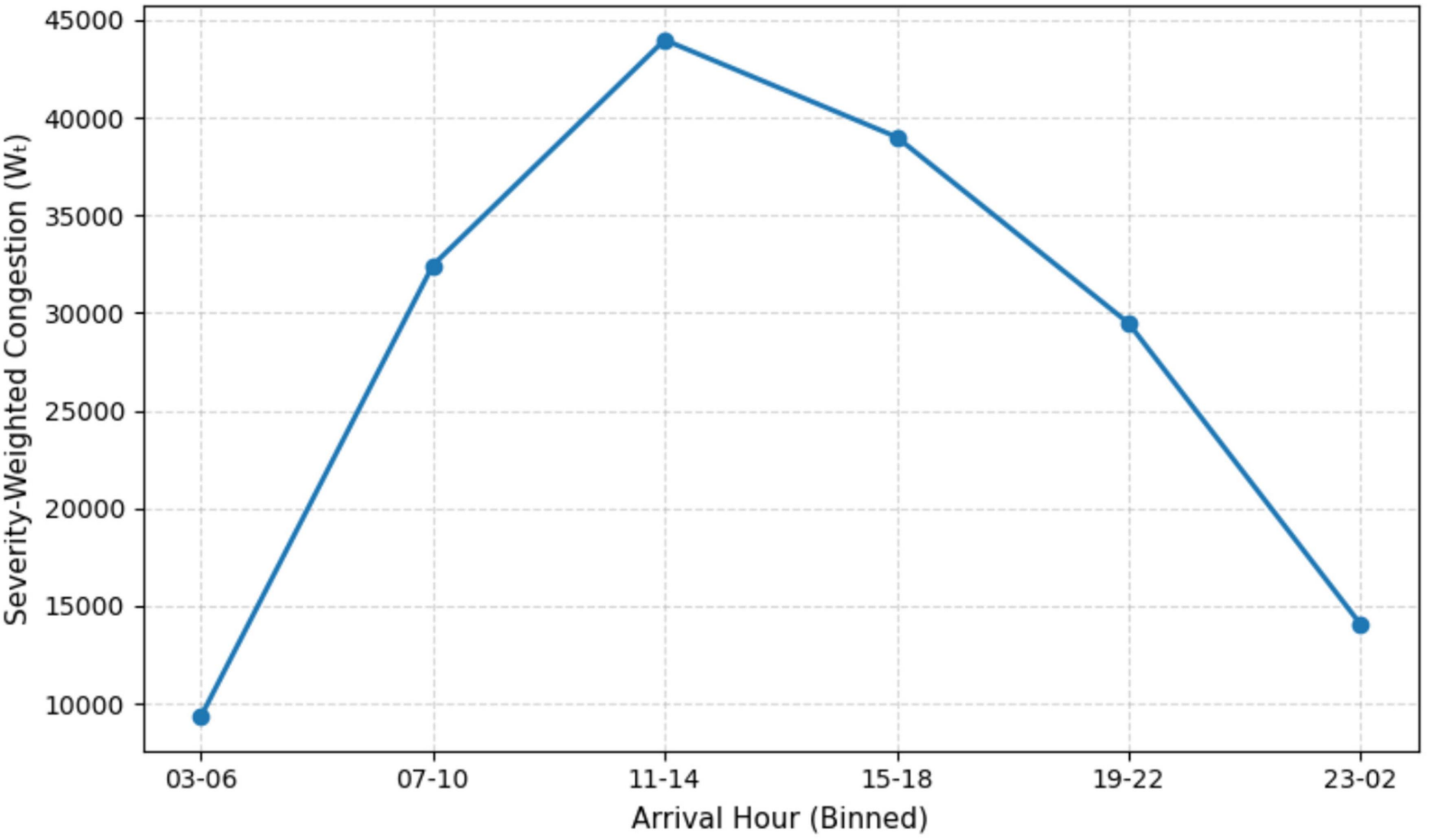
Digital twin–derived congestion profile across arrival hour intervals (dataset-2).

Figure 8 presents the congestion profile based on severity weighting for W_t obtained by hour intervals in relation to the triage dataset. The findings show that there is a higher level of congestion within mid-day hours (11:00–14:00) followed by a gradual decrease until late evenings and night-time periods. This follows a similar pattern in relation to the temporal arrival rate of patients as presented in Figure 3 using Dataset-1. Although this does not provide evidence of validity of the integrated digital twin, it demonstrates that it can reflect congestion levels through different data sets.

### 4.3 Dataset-3: outcome grounding and waiting time realism

#### 4.3.1 Waiting time distribution

**Figure 9:**
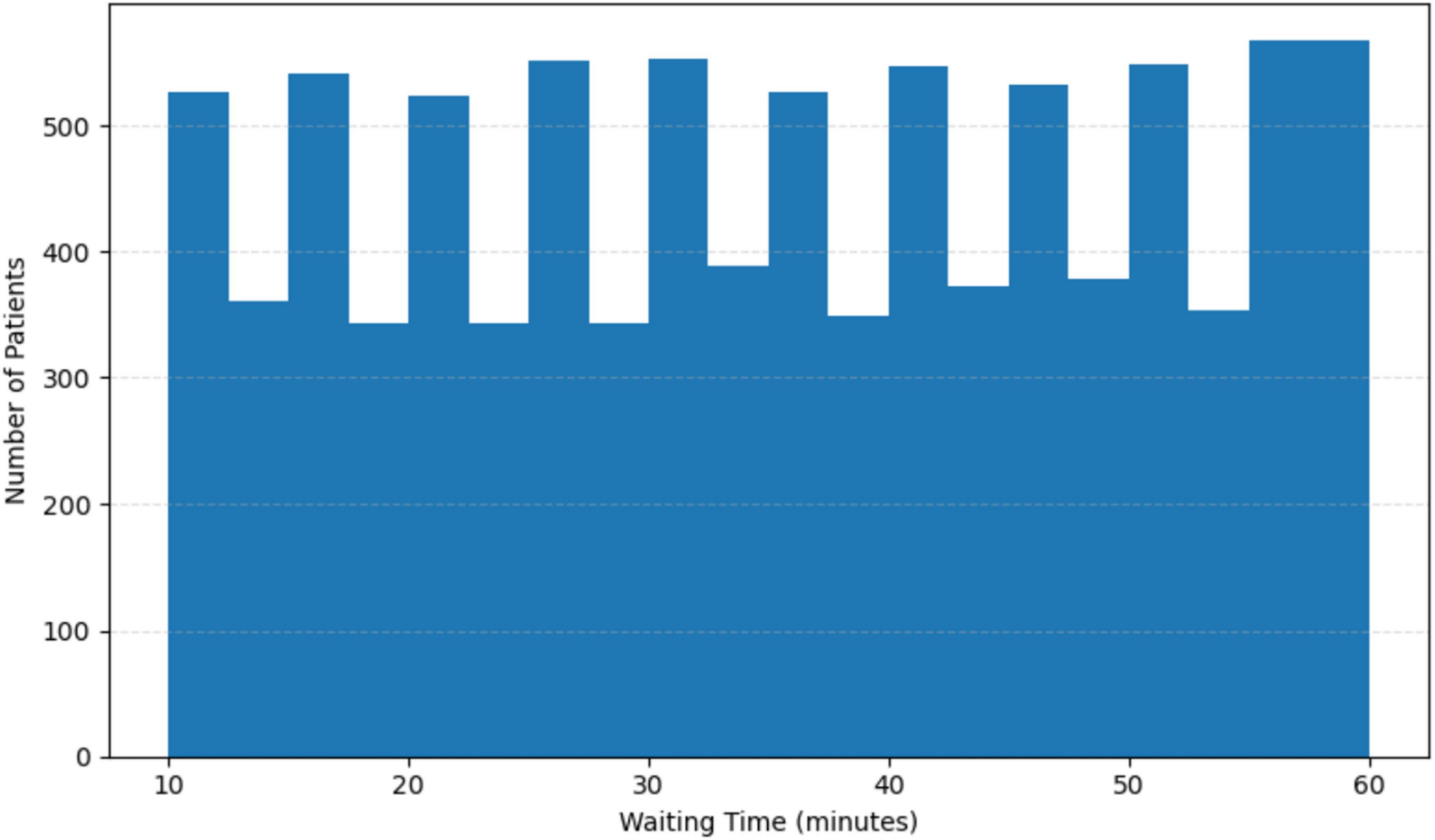
Empirical distribution of emergency department patient waiting time.

The range of this waiting time distribution is between 10 and 60 min, with an average of approximately 35 min, which reflects the average waiting times found in hospitals' emergency units under normal circumstances (Figure 9). There is no presence of extreme tails in this waiting time distribution.

**Figure 10:**
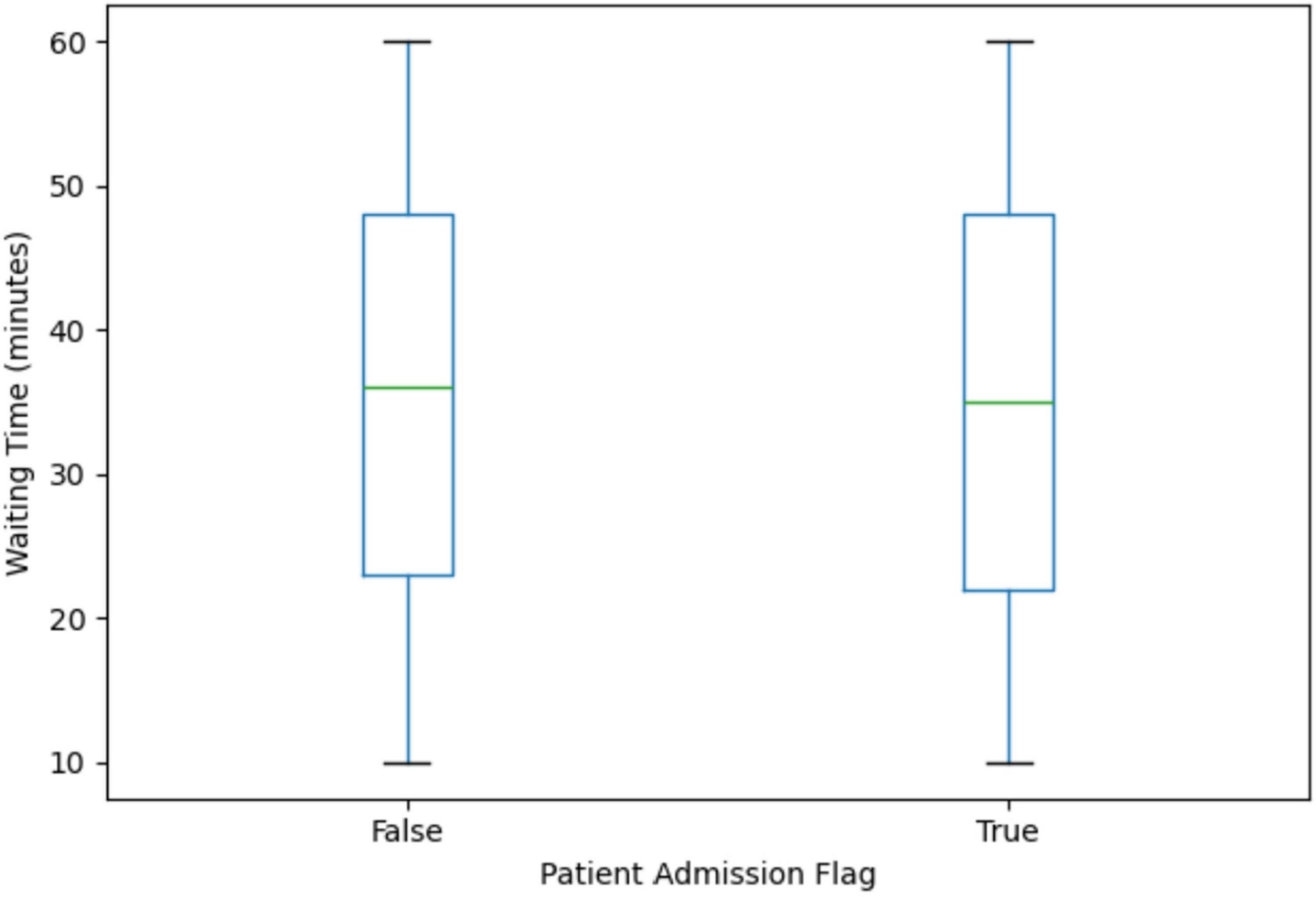
Comparison of patient waiting time by admission outcome.

Figure 10 indicates that the wait time for both admitted and non-admitted patients is almost equally distributed around 35 min. This implies that wait time behavior is fairly consistent regardless of whether the patient was admitted or not.

**Figure 11:**
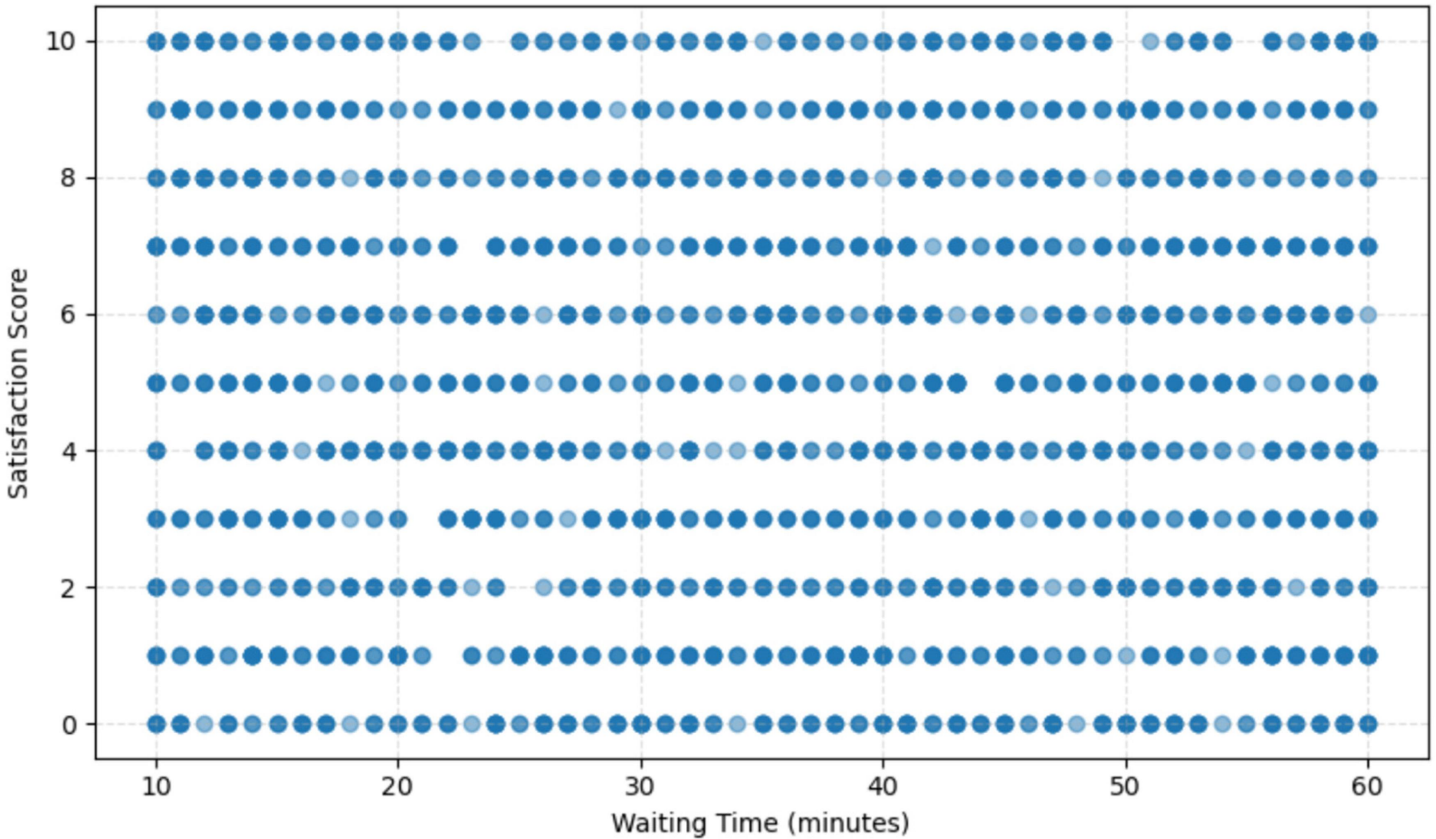
Relationship between patient satisfaction and waiting time.

A negative association between waiting time and patient satisfaction can be seen in Figure 11, meaning that long waiting times correspond to lower patient satisfaction levels. This finding is indicative of a negative correlation between waiting time and the overall patient experience. It is worth noting that such an association has been well documented in healthcare studies.

**Figure 12:**
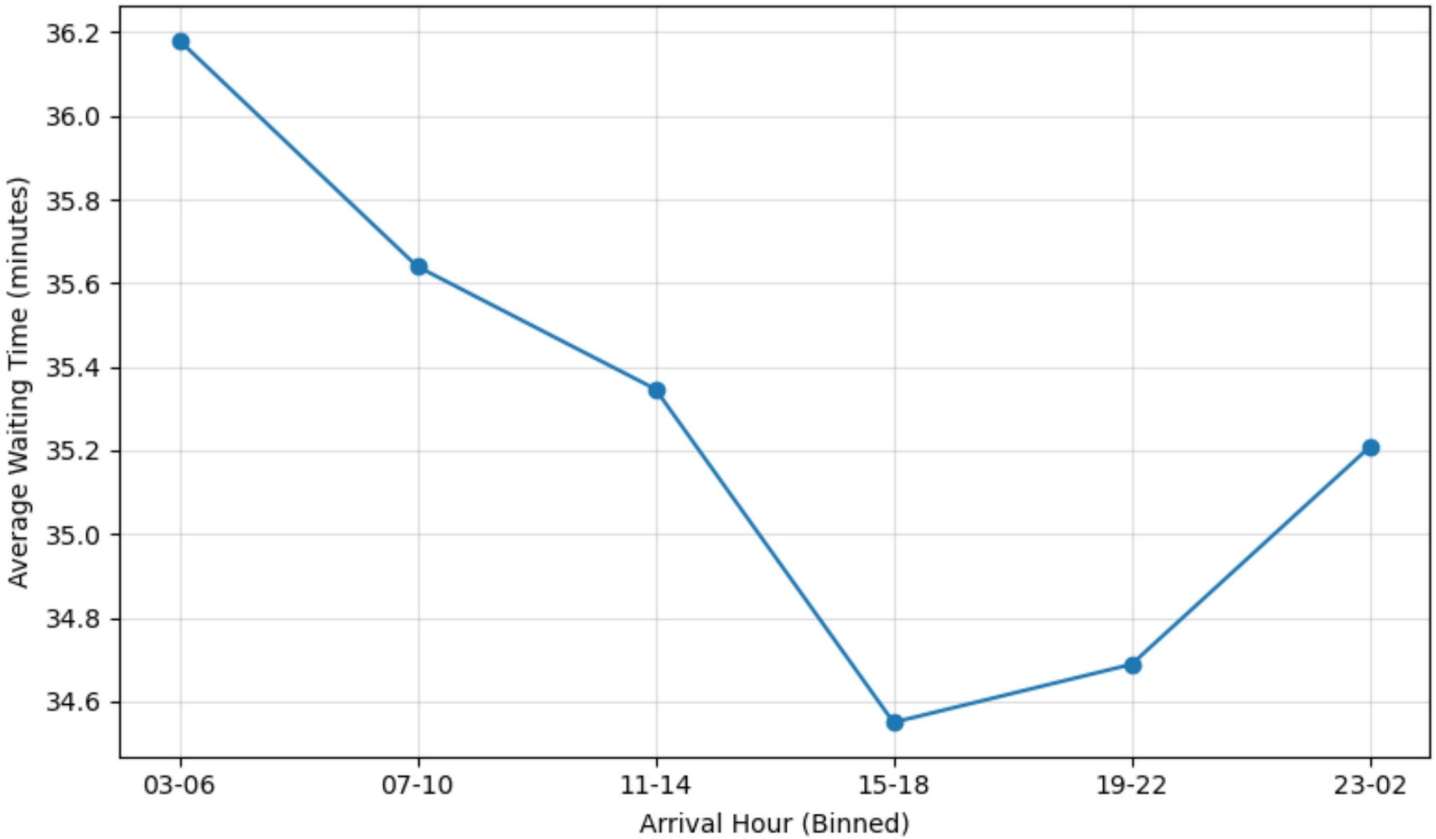
Real emergency department waiting time profile across arrival hours (dataset-3).

The profile of waiting times based on arrival times exhibits stability characteristics, with higher delays noted for arrival periods in the early mornings (03:00–06:00) and shorter waiting times noted for arrival periods in the afternoons (15:00–18:00), as depicted in Figure 12. This trend follows the same pattern of traffic congestion trends noted in Dataset-2, implying that both datasets align well in the context of time-based operations. Although this does not validate a unified digital twin, it implies that the proposed methodology can effectively capture waiting times.

These experimental observations have revealed consistent results when applied to several datasets, concerning temporal arrivals, congestion behavior based on the severity of cases, and waiting time characteristics. Temporal predictions, triage-based congestion analysis, and outcome grounding form the basis for systematic investigation into various aspects of ED performance in question. It should be noted that instead of providing a validated system for use in practical applications, these results suggest the possibility of modeling and analyzing ED operations via simulation due to the digital twin architecture. By simulating various scenarios of patient admission, one can analyze how well the system performs and optimize ED workflows accordingly. Real-time validation based on the statistics for specific hospitals is still necessary.

## 5 Conclusion

The paper describes an AI-powered digital twin architecture that is designed to assess the performance of an emergency department using the concept of temporal prediction, clinical robustness model, and outcome grounding in one architecture. Consistency in behavior is observed among the three different datasets used, implying that the suggested architecture could effectively capture certain characteristics of patient arrivals, congestion behavior, and waiting-time behavior. Temporal intelligence captures long-term arrival behavior, clinical robustness captures congestion behavior based on severity, and outcome grounding captures the correlation between waiting time and patient satisfaction.

Instead of depicting a full-fledged operational system, the proposed model gives an evidence-based viewpoint to analyze hospital process dynamics. The incorporation of various analytical elements helps in understanding how patient flow, resource allocation, and congestion dynamics affect each other in a simulation. This suggests that the combination of prediction modeling and digital twin models enables structured evaluation of health care operations, which in turn, helps to assess different scheduling approaches.

Despite the promising capabilities of the proposed approach to digital twins, further investigation is still needed to validate and implement the approach in a real-time setting. Future studies should consider incorporating real-time streams of data, utilizing more complex optimization or reinforcement learning algorithms in scheduling patients, and testing the approach in several healthcare institutions.

## 6 Future direction and limitation

It should be noted that the AI-driven digital twin model shows some consistent patterns of behavior in different data sets, suggesting its capacity to reflect several critical features related to emergency departments. At the same time, since the proposed framework relies on publicly available data sets, it requires validation using data obtained in real time directly from hospitals. Even though there is complementary information available in the data sets analyzed, it does not include any information about the actual behavior in hospitals. The next steps will be focused on obtaining real-time data sources for developing healthcare digital twins, as described in the literature (25, 26).

The study develops predictive modelling and optimisation approaches using established objective functions. It offers interpretability and operational transparency, but future research will add reinforcement learning control approaches to adapt to uncertain medical contexts. Recent advances in intelligent digital twin systems allow decision-making systems to increase autonomous operations while meeting safety criteria (26).

The system's ability to produce understandable results shows its current limitations under development. The framework maintains its operational capacity through digital twin systems, which prevent black box systems from taking over. Future studies will combine explainable and interpretable AI to show their operational details and decision-making process. The system would enhance trust and auditability to boost clinical confidence in AI-based scheduling suggestions which follows trustworthy AI standards for digital twin systems (27).

Framework assessment employed institutional datasets from a few institutional settings. Researchers must test the system at multiple hospitals and verify its performance through cross-institutional testing. Future study will examine enhanced healthcare Internet of Things system connections that allow patient monitoring, dispersed data collecting, and secure operation in intelligent hospital settings (28).

The proposed framework is valuable because its limitations demonstrate methods for developing digital twin technology for real-time healthcare decision support systems with explainable and scalable features for clinical use.

## Data Availability

The original contributions presented in the study are included in the article/Supplementary Material, further inquiries can be directed to the corresponding author/s.
